# Hedgehog-mediated gut-taste neuron axis controls sweet perception in *Drosophila*

**DOI:** 10.1038/s41467-022-35527-4

**Published:** 2022-12-19

**Authors:** Yunpo Zhao, Mohammed A. Khallaf, Emilia Johansson, Najat Dzaki, Shreelatha Bhat, Johannes Alfredsson, Jianli Duan, Bill S. Hansson, Markus Knaden, Mattias Alenius

**Affiliations:** 1 Department of Molecular Biology, Umeå University, Umeå, Sweden; 2 Max Planck Institute for Chemical Ecology, Jena, Germany; 3 Department of Zoology and Entomology, Faculty of Science, Assiut University, Assiut, Egypt; 4 Department of Biomedical and Clinical Sciences, Linköping University, Linköping, Sweden; 5 Present Address: School of Medicine, University of Maryland, Baltimore, USA; 6 Present Address: Department of Neuroscience, Max Delbrück Center for Molecular Medicine, Berlin, D-13122 Germany; 7 Present Address: Department of Microbiology and Immunology, Gothenburg university, Gothenburg, Sweden

**Keywords:** Sensory processing, Nutrient signalling, Feeding behaviour

## Abstract

Dietary composition affects food preference in animals. High sugar intake suppresses sweet sensation from insects to humans, but the molecular basis of this suppression is largely unknown. Here, we reveal that sugar intake in *Drosophila* induces the gut to express and secrete *Hedgehog* (*Hh*) into the circulation. We show that the midgut secreted Hh localize to taste sensilla and suppresses sweet sensation, perception, and preference. We further find that the midgut Hh inhibits Hh signalling in the sweet taste neurons. Our electrophysiology studies demonstrate that the midgut Hh signal also suppresses bitter taste and some odour responses, affecting overall food perception and preference. We further show that the level of sugar intake during a critical window early in life, sets the adult gut Hh expression and sugar perception. Our results together reveal a bottom-up feedback mechanism involving a “gut-taste neuron axis” that regulates food sensation and preference.

## Abstract (web summary)

Food regulates taste perception, but the underlying molecular mechanisms are not clear. Here, the authors reveal that sugar intake in Drosophila induces the gut to secrete Hedgehog into the circulation that suppresses sweet taste, sugar perception and preference.

## Introduction

Sugar is a major energy source for most animals, and thus sweet taste typically mediates attraction (i.e., has a positive valence). This innate drive to consume sugar must be fine-tuned to avoid insulin resistance and type 2 diabetes. Sugar metabolism and energy status in the muscles, gut, and adipose tissue regulate insulin release^1^, which then balances the metabolic requirements of the periphery. In mice and humans, dopaminergic mesolimbic neurons reshape sugar responses and their activation produces an intense desire for sugar^2^. Accumulating evidence suggests sugar reward is suppressed by insulin, which passes through the blood brain barrier and suppresses dopamine pathways^3,4^. Therefore, insulin resistance in the reward circuits leads to sugar overconsumption and obesity in humans^5–7^. Thus, in both mice and humans, peripheral sugar metabolism can act through insulin to reduce the central drive for sugar consumption, reducing sugar intake.

In *Drosophila*, the insulin producing cells (IPCs) are localized in the brain and thus communicate central energy status^8^. IPCs are regulated by *Gr43a* taste receptor-expressing neurons in the fly brain that sense internal sugar status. They then promote feeding in food-deprived flies and suppress feeding in fed flies^9,10^. Energy status also regulates the activity of dopaminergic taste interneurons that control food consumption^11^, suggesting that feeding in flies may also be regulated at the level of taste perception. Ingested sugar also regulates sweet perception in *Drosophila*^12^, with low sugar intake increasing sweet sensation and high sugar intake suppressing sweet perception^12–15^. This suggests that the periphery in *Drosophila* uses signals other than insulin to balance sugar intake. However, the circuitry and signals that communicate peripheral sugar metabolism to regulate sweet taste sensitivity remain unknown.

Sugar suppression of sweet sensation also occurs in mice and humans^16^. We therefore hypothesized that the same sweet suppressive signal may regulate sugar metabolism in both *Drosophila* and mice. In both species, Hedgehog (Hh) signalling regulates sugar metabolism^17,18^. Interestingly, dysfunctional sugar metabolism increases the release of the vertebrate Hh orthologue Sonic hedgehog (Shh) into the blood^19^. In *Drosophila* larva, the midgut secretes Hh into the haemolymph^20^. After its release, Hh binds and inhibits its receptor Patched (Ptc)^21–23^, activating a signalling cascade that induces Hh target genes^24^. In adult flies and in mice, Hh signalling regulates odorant receptor transport and olfaction^25,26^. This sensory regulation, together with the link to sugar metabolism, suggested to us that circulating Hh may be the signal regulated by dietary sugar that is responsible for regulating sweet perception.

Here, we reveal that sugar suppresses sweet sensation in fed flies. We further reveal that Hh from the midgut mediates responses to dietary sugar. Our results show that sugar regulates midgut Hh expression, but it does this differently in immature and mature flies. This suggests the existence of a critical window in sugar perception. Our mechanistic analysis revealed that the endocrine Hh signal suppresses a local autocrine Hh signal in olfactory and taste sensory neurons. We also show that suppression of the autocrine pathway alters both taste and odour responses, suppressing sweet sensation and preference.

## Results

### Dietary sugar suppresses sweet taste perception in fed flies

**Fig. 1:**
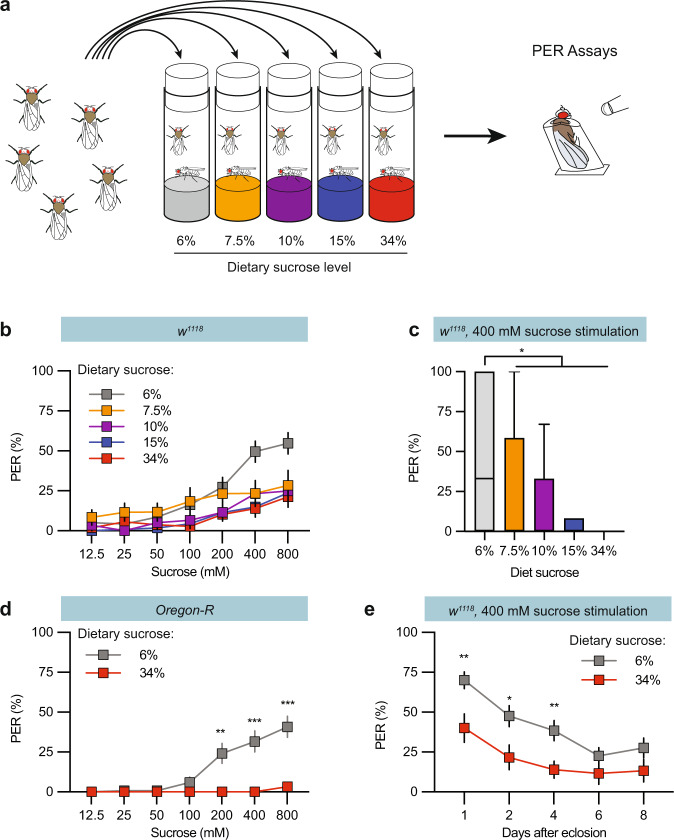
Regulation of sweet taste responses by dietary sugar. **a** Experimental procedure schematic. Newly eclosed flies were shifted to diets supplemented with various levels of sucrose. Proboscis Extension Response (PER) assays were performed four days later. **b** Plot of sugar-evoked PER in *w*^*1118*^ males reared on the indicated diets (colour-coded). The y-axis shows PER %. The x-axis shows sucrose concentration in the stimulation solution. **c** Box plot showing the PER response to 400 mM sucrose stimulation of male flies fed the indicated sugar diets. Median (middle line) is depicted, and whiskers indicate Tukey. **d** Plot of sugar-evoked PER in *Oregon-R* males reared on the indicated diets. **e** Plot of sugar-evoked PER in 1, 2, 4, 6, and 8-day old *w*^*1118*^ males reared on the indicated diets. Data are presented as means ± SEM. *n* = 20–40 flies. PER data were non-normally distributed. Statistical analyses were performed via either two-tailed Mann-Whitney tests (**d** and **e**) or Kruskal-Wallis H-tests with Dunn’s tests for multiple comparisons (**c**). *p < 0.05; **p < 0.01; ***p < 0.001. Source data are provided as a Source Data file.

An adult *Drosophila* extends its proboscis when presented with a nutritious stimulus. This proboscis extension response (PER) functions as a proxy for taste perception (Fig. 1a). Previous studies have reported that high dietary sugar suppresses the sugar PER^12,14^, but these studies only tested starved animals. We asked whether excess dietary sugar would similarly suppress the sugar PER in fed male flies (Fig. 1b, c). Indeed, when we fed flies a standard diet supplemented with a lower amount of sugar, they exhibited a higher PER than flies fed more sugar (i.e., the standard 6% sucrose diet supplemented to 7.5, 10, 15, or 34% sucrose) (Fig. 1b, c). OregonR flies showed an even stronger suppression than the white background strain (Fig. 1d).

In previous studies with starved flies, sweet suppression occurred after several days of high sugar consumption^12,14^. When fed flies were shifted from a diet containing 6% sugar to one containing 34% sugar directly after eclosion, we observed an immediate and significant suppression in the PER for the first 4 days and a consistently lower trend in the responses thereafter compared to flies fed a 6% sugar diet (Fig. 1e). Thus, increased sugar ingestion suppresses sweet perception in both fed and starved flies, but with different onsets.

### Midgut-derived Hh suppresses sweet taste perception

**Fig. 2:**
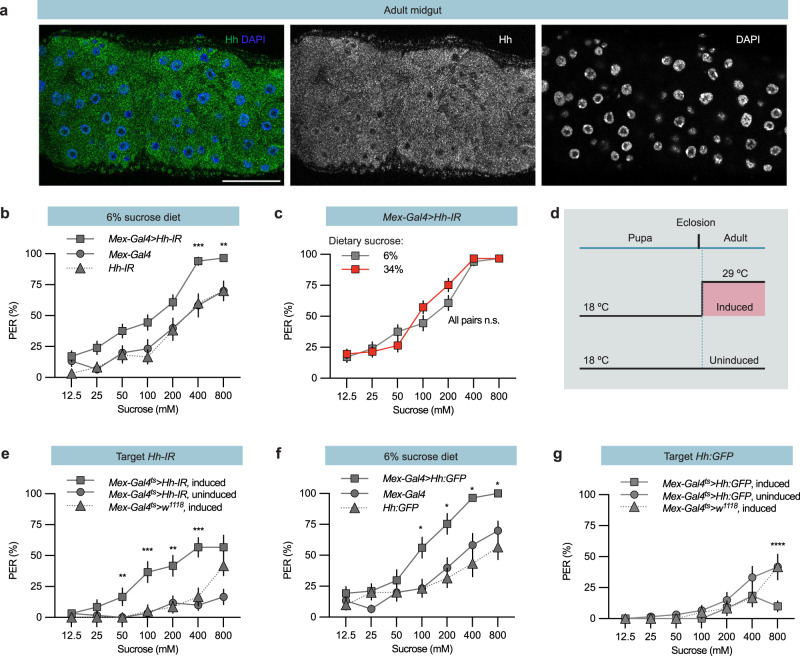
Gut Hh regulates sweet taste responses. **a** Representative confocal image of a male adult *w*^*1118*^ midgut. Anti-Hh staining is shown in green, DAPI stains nuclei in dark blue in the merged image. Scale bar, 50 µm. **b** Knocking down *Hh* in gut enterocytes (*Mex* > *Hh-IR)* produced a significant increase in sucrose-induced PER. **c** *Mex* > *Hh-IR* flies showed a similar PER % when reared on the indicated sugar diets. **d** TARGET experiment schematic. Flies were maintained at 18 °C to restrict Gal4 activity. Newly eclosed flies were shifted to 29 °C for 4 days to induce gene expression or maintained in the uninduced state at 18 °C. **e** Plot showing a four-day TARGET induction of *Hh-IR* expression in the enterocytes of adult males leads to a significantly higher level of PER than that observed in the uninduced controls. **f** Hh-GFP overexpression in enterocytes (*Mex* > *Hh-GFP*) increased PER. **g** A four-day induction of *UAS-Hh-GFP* expression in the enterocytes of adult males significantly decreased PER compared to the controls. Data are presented as means ± SEM. n = 20–40 flies Statistical analyses were performed via either two-tailed Mann-Whitney tests (**d**) or Kruskal-Wallis H-tests with Dunn’s tests for multiple comparisons (**b**, and **e–g**). *p < 0.05; **p < 0.01; ***p < 0.001; ****p < 0.0001. Source data are provided as a Source Data file.

Next, we set out to identify the nature of the signal that suppresses sweet perception. We hypothesized that the signal should be involved in sugar metabolism and regulate chemosensation in both *Drosophila* and mice. Hh signalling regulates sugar metabolism^17,18^ and regulates odorant receptor transport and olfaction in both species^25,26^. This sensory regulation, together with the link to sugar metabolism, made Hh our main candidate. Interestingly, Hh expression in the midgut is regulated according to metabolic state in *Drosophila* larvae^27^. This made us ask whether the adult gut also expresses Hh. Immunohistochemistry with a previously established Hh antibody^28^ showed that the large polyploid midgut enterocytes express Hh (Fig. 2a). Thus, we hypothesised that sugar levels in the gut regulate sugar perception via Hh signalling.

To determine whether midgut Hh regulates sugar perception, we used the midgut-specific driver *Mex-Gal4*^29,30^ (Fig. S1a, b) to express an Hh inverted repeat (Hh-IR) and knock down Hh in enterocytes. Compared to *Mex-Gal4* control flies, depletion of Hh in the midgut increases the sugar PER of flies fed the low sugar standard diet (6%, Fig. 2b). This suggests midgut Hh suppresses sugar perception. Interestingly, when we shifted these *Mex-Gal4* > *Hh-IR* flies to the 34% sugar diet, the normal suppression of the PER was lost (Fig. 2c).

Midgut-derived Hh is a major regulator of *Drosophila* development^27^. Since male midguts do show small growth-related changes in adults^31^, the suppression we observed could be due to an Hh-related change in midgut enterocyte number. When we counted the enterocytes, however, we did not observe any difference between control and Hh knock down flies (Fig S2a, b), indicating that the suppression we observed is not due to a developmental effect. To further rule out any developmental effect of the midgut Hh knock down, we used the TARGET system^32^. This technique uses a ubiquitously expressed temperature-sensitive Gal80 (Gal80^ts^) to repress Gal4’s activation of UAS at 18 °C but permit it at 29 °C (Fig. 2d). Shifting TARGET *Mex-Gal4* > *Hh-IR* flies from 18 °C to 29 °C immediately after eclosion limits the Hh knock down to the adult stage. The adult Hh knock down increased the sugar PER (Fig. 2e), confirming that Hh depletion in adult enterocytes affects sugar sensation and taste suppression.

Surprisingly, Hh overexpression in the midgut (*Mex-Gal4* > *UAS-Hh:GFP*) did not suppress but rather increased the sugar PER (Fig. 2f). To determine whether this increase in sweet sensation arises from a change during development, we again employed the TARGET system to restrict *Hh:GFP* overexpression to adult flies. Compared to temperature-matched controls, those overexpressing Hh in their gut exhibited a suppression of the PER (Fig. 2g). Control flies expressing only TARGET *Mex-Gal4* shifted to 29 °C showed no changes in PER (Fig. 2g). This difference indicates that Hh expression affects sweet taste neuron development and that adult Hh expression affects sweet sensory neuron responses. Thus, the midgut Hh signal in adult flies is both necessary and sufficient to suppress sweet perception.

### Sugar intake controls midgut Hh expression and forms a sugar memory

**Fig. 3:**
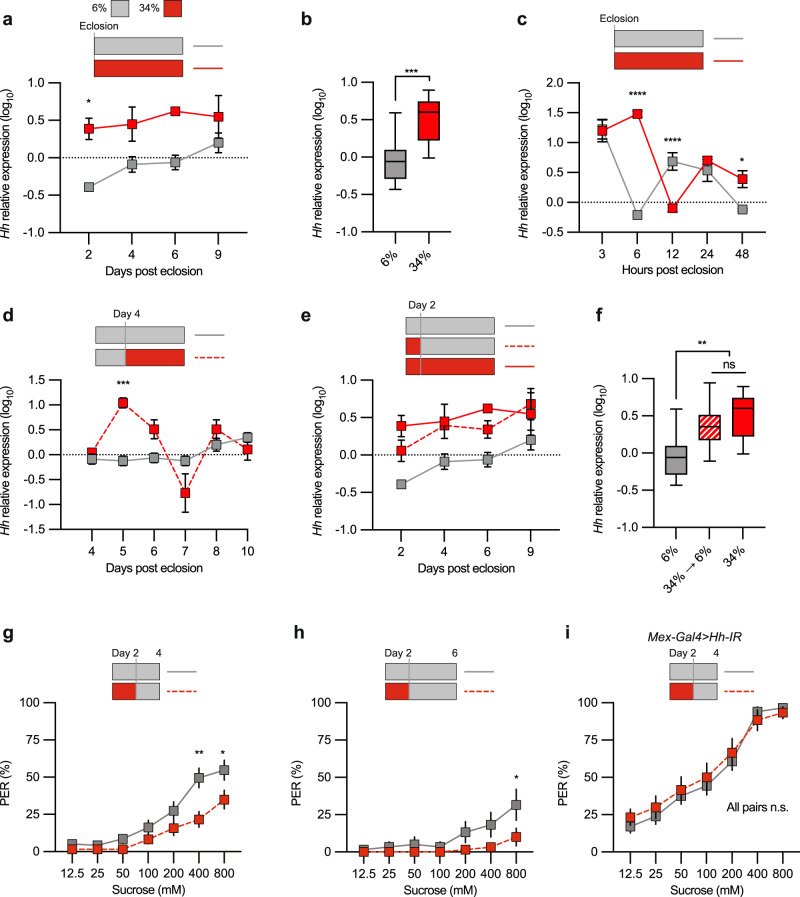
Regulation of gut Hh expression by dietary sugar. **a** Plot of a qPCR analysis *w*^*1118*^ control flies *Hh* gut expression after a shift to either 6% or 34% sugar diet after eclosion. **b** A box plot showing the total *Hh* gut expression during the days 2–9 in a. The middle line indicates the median, and the whiskers indicate minimum and maximum values. **c** 6% or 34% sugar diets influence on gut *Hh* expression during the first 48 hours post eclosion. **d** midgut *Hh* expression in flies on the 6% sugar diet for 4 days and then shifted to 6% or 34% sugar diets. (**e**) Midgut *Hh* expression in flies on 6% or 34% sugar diets for 2 days and shifted to 6% or 34% sugar diets. **f** A box plot showing the total gut *Hh* expression during the days 2–9 for each diet in e. The middle line indicates the median, and the whiskers indicate minimum and maximum values. **g** PER results of *w*^*1118*^ flies on 6% or 34% sugar diets for 2 days and shifted to the 6% diet for 2 days. **h** PER results of *w*^*1118*^ flies on 6% or 34% sugar diets for 2 days and shifted to the 6% diet for 4 days. **i** PER results of *Mex-Gal4* > *Hh-IR* flies on the 6% or 34% sugar diets for 2 days and shifted to 6% diet for 2 days. Data are presented as means ± SEM. **a**–**f** *n* = 3–7 bioreplicates. **g**–**i** *n* = 20–40 flies. Statistical analysis was performed via *t*-test (**b**), one-way ANOVA with the Tukey correction (**f**), two-way ANOVA with the Sidak correction (**a**, **c**, and **d**), or two-tailed Mann-Whitney tests (**g**–**i**). *p < 0.05; **p < 0.01; ***p < 0.001; ****p < 0.0001. Source data are provided as a Source Data file.

To determine whether dietary sugar regulates Hh midgut expression, we measured Hh expression in the dissected adult midgut via quantitative polymerase chain reaction (qPCR). Flies on the standard 6% sugar diet showed low Hh expression (Fig. 3a, b). Shifting flies upon eclosion from the 6% sugar maintenance diet to the 34% sugar diet increased midgut *Hh* expression (Fig. 3a, b), indicating that Hh expression is related to dietary sugar intake.

To clarify the timing of sugar’s effect on Hh gene regulation, we quantified Hh expression at pre-determined times after a dietary shift. Flies exposed to a 34% sugar diet directly after eclosion exhibited a drastic overshoot at hour 6 in Hh expression, followed by a dip and a rebound to the adult Hh level by hour 48 (Fig. 3c). This nearly immediate upregulation of Hh suggests that an extreme change in the sugar content of a fly’s food during from larva (6%) to adult (34%) directly alters Hh expression. Curiously, flies raised on the standard sugar diet already showed increased midgut Hh expression by hour 3 (Fig. 3c), and then experienced a dip and rebound, eventually settling into a level significantly lower than that of high sugar diet-fed flies at hour 48 (Fig. 3c). Together, these observations imply that the initial post-eclosion feeding induces dynamic changes in Hh expression that determine basal Hh expression in the adult.

We next asked whether this switch only occurs if the dietary change takes place early in life. When we fed adult flies the 6% maintenance diet for 4 days and then shifted them to a 34% sugar diet, we observed an initial increase in Hh expression and a prolonged oscillation that lasted for 6 days (Fig. 3d). Nevertheless, Hh levels did return to the 6% base line even if the flies were maintained on a 34% diet (Fig. 3d). This suggests that sugar-induced changes in Hh expression early in life may be permanent. To further determine whether early exposure to a high sugar diet sets the adult Hh expression level and to better define this apparent critical window for feeding, we shifted newly eclosed flies to a 34% sugar diet for two days before returning them to the standard diet for a week. The switched flies expressed levels of Hh comparable to flies maintained on high dietary sugar for the entire period (Fig. 3e, f). Therefore, our observations showed that dietary sugar content during early adult life imprints on Hh expression.

To determine whether the early critical window also affects taste perception, we again shifted newly eclosed flies to a 34% sugar diet for two days before returning them to the standard diet for two respective four more days. Interestingly, these flies showed a suppressed sugar PER like controls maintained on high sugar (Fig. 3g, h). We did not observe this persistent suppression in gut Hh knock down flies (Fig. 3i). Together, our results reveal that dietary sugar regulates Hh midgut expression and that the level of sugar intake early in life has long-lasting effects on both Hh levels and sugar perception, indicating the existence of a sugar regulatory critical window.

### Sugar induces Hh midgut secretion into the haemolymph

**Fig. 4:**
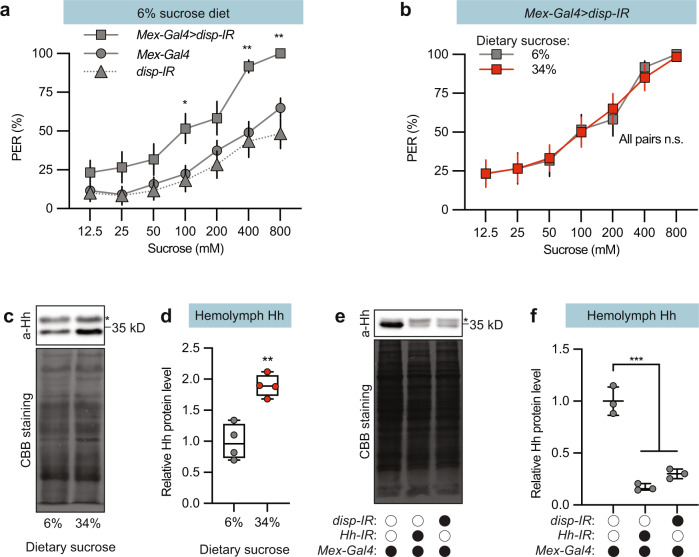
Gut-derived Hh is secreted into the haemolymph. **a** Knock down of *disp* in gut enterocytes (*Mex* > *disp-IR)* significantly increased sucrose-induced PER. **b** *Mex* > *disp-IR* flies showed a similar PER % when reared on the indicated sugar diets. **c** Anti-Hh immunoblot of total protein from the hemolymph of *w*^*1118*^ males reared on the indicated diets. The asterisk indicates a nonspecific band. Staining with Coomassie brilliant blue (CBB) serves as a loading control (below). **d** Plot showing the quantification of circulating Hh levels in **c**. *n* = 4. Median (middle line) is depicted, and whiskers represent minimum to maximum. **e** Anti-Hh immunoblot of total protein from the hemolymph of the indicated genotypes. Flies were reared on the 34% sugar diet. The asterisk indicates a nonspecific band. Staining with Coomassie brilliant blue (CBB) serves as a loading control (below). **f** Plot showing the quantification of circulating Hh levels in (3). The Hh band was normalized to the CBB stained total protein. *n* = 3. Data are presented as means ± SEM (**a**, **b**). *n* = 20–40 flies or means ± SD (**d**, **f**). Statistical analyses were performed via Kruskal-Wallis H-tests with Dunn’s tests for multiple comparisons (**a**), two-tailed Mann-Whitney tests (**b**), two-tailed *t*-test (**d**), or one-way ANOVA with Tukey correction (**f**). *p < 0.05; **p < 0.01; ****p < 0.0001. Source data are provided as a Source Data file.

In *Drosophila* larvae, Hh from the midgut is secreted into the haemolymph^27^. Hh secretion in both flies and mice is a complex process involving cleavage, cholesterol modification, and palmitoylation^33,34^. In *Drosophila* larvae, *dispatched* (*disp*) is required for cholesterol modification and also midgut Hh secretion into the haemolymph^20,27^. We therefore asked whether disp is also required for the endocrine Hh regulation of sweet taste perception. Indeed, like *Mex-Gal4* > *Hh-IR* flies, *Mex-Gal4* > *disp-IR* flies maintained on the 6% sugar diet exhibited increased PER (Fig. 4a) and the sugar induced suppression was abolished (Fig. 4b), indicating that sugar intake regulates both midgut Hh expression and secretion.

To determine whether Hh secreted from the midgut reflects sugar intake, we collected haemolymph from 4-day-old flies and performed a western blot. The blot showed two bands: a 32-kD band, which correlates with cleaved, active Hh, as well as a slightly larger band (Fig. 4c). Zhang et al. showed that RNAi-mediated Hh knock down depleted the lower band but did not affect the upper band^35^, suggesting that the upper band represents nonspecific binding. We also found that Hh knock down in enterocytes using *Mex-Gal4* > *Hh-IR*, only reduced the lower band (Fig. 4e), suggesting midgut enterocytes are the major source of circulating Hh. *Mex-Gal4* > *disp-IR* flies also showed reduced Hh in the circulation (Fig. 4e, f), consistent with regulated Hh secretion. There was also more Hh in the haemolymph of flies on the 34% sugar diet compared to flies on the standard 6% sugar diet (Fig. 4c, d). These results are consistent with the hypothesis that sugar induces midgut Hh expression and secretion.

### Midgut Hh targets taste and olfactory sensory neurons

**Fig. 5:**
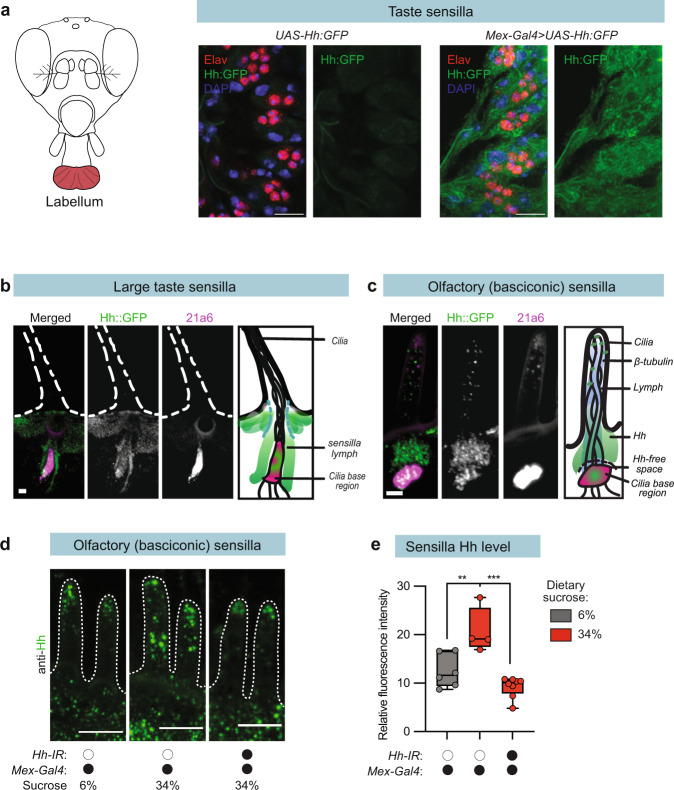
Gut-derived Hh is transported to the antenna and labellum. **a** Visualization of gut-expressed Hh:GFP (*Mex-Gal4* > *Hh:GFP*) in the labellum. Anti-Elav staining is in red, DAPI in blue. **b** Taste sensilla in *Mex-Gal4* > *Hh:GFP* flies, Hh:GFP (GFP, green) and cilia base (21A6, magenta). **c** Olfactory sensilla in *Mex-Gal4* > *Hh:GFP* flies, Hh:GFP (GFP, green), and cilia base (21A6, magenta). **d** Visualization of endogenous Hh localization in basiconic sensilla, Hh (green). **e** Plot showing the relative fluorescence intensity of anti-Hh staining in **c**. Median (middle line) is depicted, and whiskers show minimum to maximum. Statistical significance determined via one-way ANOVA. *n* = 6–8 sensilla cell clusters **p < 0.01; ***p < 0.001. Scale bar for a, 10 µm; for **b** and **c**, 1 µm; for d, 5 µm. Source data are provided as a Source Data file.

To visualize Hh transport from the midgut and identify possible target cells, we expressed *UAS-Hh:GFP* under the control of *Mex-Gal4* and visualized the resulting GFP expression. We observed a clear Hh:GFP signal in both the labellum and the antennae (Fig. 5a–c), thus showing that Hh is transported from the midgut into the peripheral taste and olfactory sensory organs. In flies, olfactory and taste sensilla contain a lymphatic fluid similar that it is distinct from the circulation and surrounds the ciliated dendrites of the sensory neurons^36^. Interestingly, our histology showed that Hh:GFP staining localized to the sensillar lymph at the base of both taste and olfactory sensilla (Fig. 5b, c). Upon closer inspection, we observed that the midgut-derived Hh:GFP localized to the tips of the dendrites within the lymph compartment (Fig. 5b, c, S3). Tight junctions between glial (support) cells and the sensory neurons separate the taste and olfactory sensillum lymph from the hemolymph^36^. In taste sensilla, Hh:GFP was also associated with glial (support) cells (Fig. 5b), suggesting that glia transport circulating Hh into the sensillar lymph.

We further performed immunohistochemistry using the Hh antibody^28^. Although taste and trichoid sensilla stain in general poorly^25^, we did observe clear localization of Hh in the large basiconic sensilla of the antenna (Fig. 5d). When we quantified sensillar Hh levels, we found that Hh uptake into large basiconic sensilla increased with dietary sugar (Fig. 5e) and that knock down of Hh expression in the midgut (*Mex-Gal4* > *Hh-IR*) eliminated sugar-induced Hh uptake (Fig. 5d, e). taken together these results suggest that an endocrine Hh signal originating from the midgut might regulate a Hh pathway in taste and olfactory sensory neurons.

### High sugar intake inhibits Hh signalling in sweet taste neurons

**Fig. 6:**
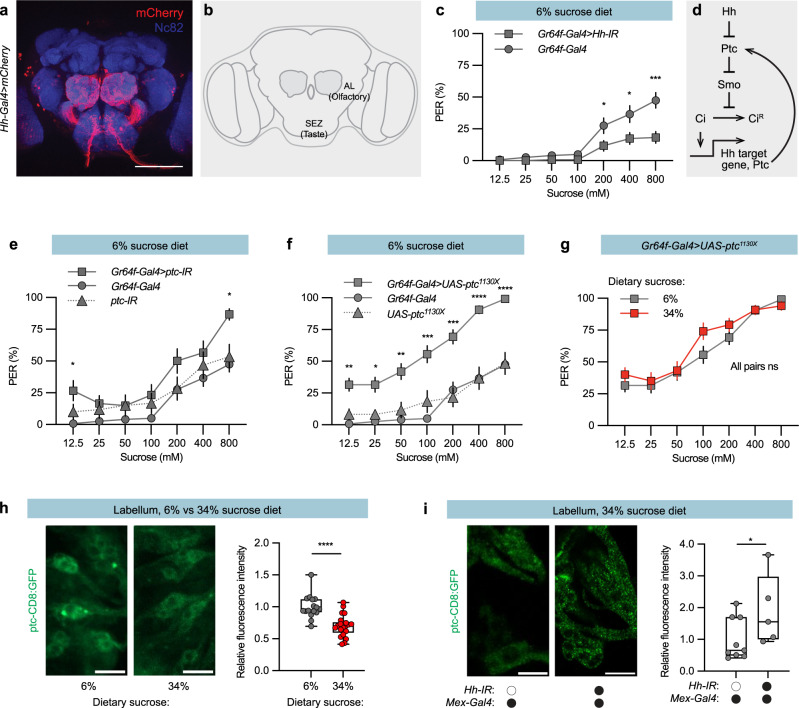
Dietary sugar suppresses autocrine Hh signalling in sweet taste neurons via an endocrine Hh signal. **a** Expression of *Hh-Gal4* (*mCherry*) in a male adult brain. Anti-Nc82 staining is shown in blue. Scale bar, 100 μm. **b** *Drosophila* brain schematic. The antennal lobe (AL) and the suboesophageal zone (SEZ) is the brain regions where olfactory respective taste sensory neurons connect to their projection neurons. **c** Plot showing reduced PER in *Gr64f* > *Hh-IR* flies maintained on a 6% sugar diet. **d** Schematic of the Hh pathway. Hh, Hedgehog; Ptc, Patched; Smo, Smoothened; Ci, Cubitus interruptus; Ci^R^, cleaved form of Ci, which functions as a transcriptional repressor. **e** Knock down of the Hh receptor Patched (Ptc) in Gr64f sweet sensory neurons increased the PER. **f** Expression of a dominant negative version of the Hh receptor Patched (*Gr64f-Gal4* > *Ptc*^*1130X*^) in sweet sensory neurons increased the PER. **g** *Gr64f-Gal4* > *Ptc*^*1130X*^ flies showed a similar PER % when reared on the indicated sugar diets. **h** Left, representative confocal images of *Drosophila* labella. Right, a box plot showing *ptc-CD8:GFP* reporter expression in the labellum. Flies maintained on the 34% sugar diet show lower *ptc-CD8:GFP* reporter expression than flies maintained on the 6% sugar diet. Scale bar, 10 μm. *n* = 16 respective 22 sensilla clusters from 4 respective 6 flies. Median (middle line) is depicted, and whiskers indicate minimum to maximum. **i** Left, representative confocal images of *Drosophila* labella. Right, a box plot showing the depletion of intestinal Hh increases *ptc-CD8:GFP* reporter expression in the labellum. Scale bar, 10 μm. *n* = 9 respective 5 sensilla clusters in 3 animals each. Median (middle line) is depicted, and whiskers indicate minimum to maximum. Data are presented as means ± SEM (**c** and **e**–**g**). **c**, **e**, **f**, **g** *n* = 20–40 flies. Statistical analyses were performed via either two-tailed Mann-Whitney tests (**c**, **g**), Kruskal-Wallis H-tests with Dunn’s tests for multiple comparisons (**e**, **f**), or one-tailed *t*-tests (**h**, **i**). *p < 0.05; **p < 0.01; ***p < 0.001; ****p < 0.0001. Source data are provided as a Source Data file.

It is previously shown that an autocrine Hh signal regulates the ciliary transport of odorant receptors and enables olfactory responses^25,26^. Hh-Gal4^37^ was also expressed in the olfactory sensory neuron projections to the antennal lobe and additionally in the taste neuron projections in the suboesophageal zone (sez) (Fig. 6a, b), suggesting that taste sensory neurons also have an autocrine Hh signal. To determine whether this local Hh source regulates sweet taste perception, we used *Gr64f-Gal4* to express *Hh-IR* in mature sweet sensory neurons^38^. The resulting *Gr64f-Gal4; Hh-IR* knock-down flies showed a reduced sugar PER (Fig. 6c), indicating that the normal sweet perecption requires this local autocrine Hh signal. Hh binds and inhibits Patched (Ptc), but unbound Ptc inhibits signalling downstream of Hh (Fig. 6d). Ptc knock-down flies also showed increased sugar PER (Fig. 6e), consistent with that local Hh signaling regulate sweet perception.

To identify the step at which step the endocrine Hh signal might suppresses the local Hh pathway, we first expressed a dominant-negative version of the Hh receptor *Ptc* (*UAS-Ptc*^*1130X*^). *Ptc*^*1130X*^ lacks the final 156 amino acids of its cytoplasmic tail, making it capable of constitutive activation of the Hh pathway^39^. Interestingly, *Gr64f-Gal4; UAS-Ptc*^*1130X*^ flies showed a similar phenotype as the midgut Hh knock down flies, increased PER on the 6% sugar diet (Fig. 6f), and no suppression of PER on the 34% sugar diet (Fig. 6g). The fact that constitutive activation of Hh signalling in sweet taste neurons blocked the sugar-induced suppression of sweet perception, strongly suggests that both sugar and the midgut Hh signal suppresses autocrine Hh signalling in taste neurons at or above the level of Ptc.

To determine whether the endocrine Hh signal suppresses taste sensory neuron Hh signalling, we generated and visualised Hh signalling with a reporter. Ptc suppresses the activation of the transcription factor Ci (Fig. 6d)^24^, but Ptc itself is also a Ci-regulated gene. Hh activity can thus, be visualized with a promoter fusion between the first kb upstream of the *Ptc* gene that contains three Ci binding motifs and *CD8:GFP*^40^. The resulting Ptc-CD8:GFP reporter showed high expression in the labellar taste neurons when flies were maintained on the 6% sugar diet and lower expression when flies were maintained on the 34% sugar diet (Fig. 6h). Sugar also suppressed Ptc-CD8:GFP reporter expression in the antenna (Fig. S4a). These results support the hypothesis that sugar suppresses the Hh signalling pathway that is otherwise continuously active in both taste and olfactory sensory neurons. Consistent with the hypothesis that the sugar-induced suppression of the Ptc-CD8:GFP reporter was a result of the endocrine Hh signal, we found that the sugar induced suppression of the Ptc-CD8:GFP reporter was lost in *Mex-Gal4* > *Hh-IR* flies (Fig. 6h and Fig. S4c). A similar Ptc-CD8:GFP reporter with mutated Ci binding motifs was also insensitive to sugar and midgut Hh knock down (Fig. S4e–h), indicating that the sugar-induced reporter suppression requires Ci. Together, these results indicate midgut-derived Hh inhibits taste perception and sensory neuron Hh signalling.

### Hh secreted from the midgut suppresses taste and odour sensation

**Fig. 7:**
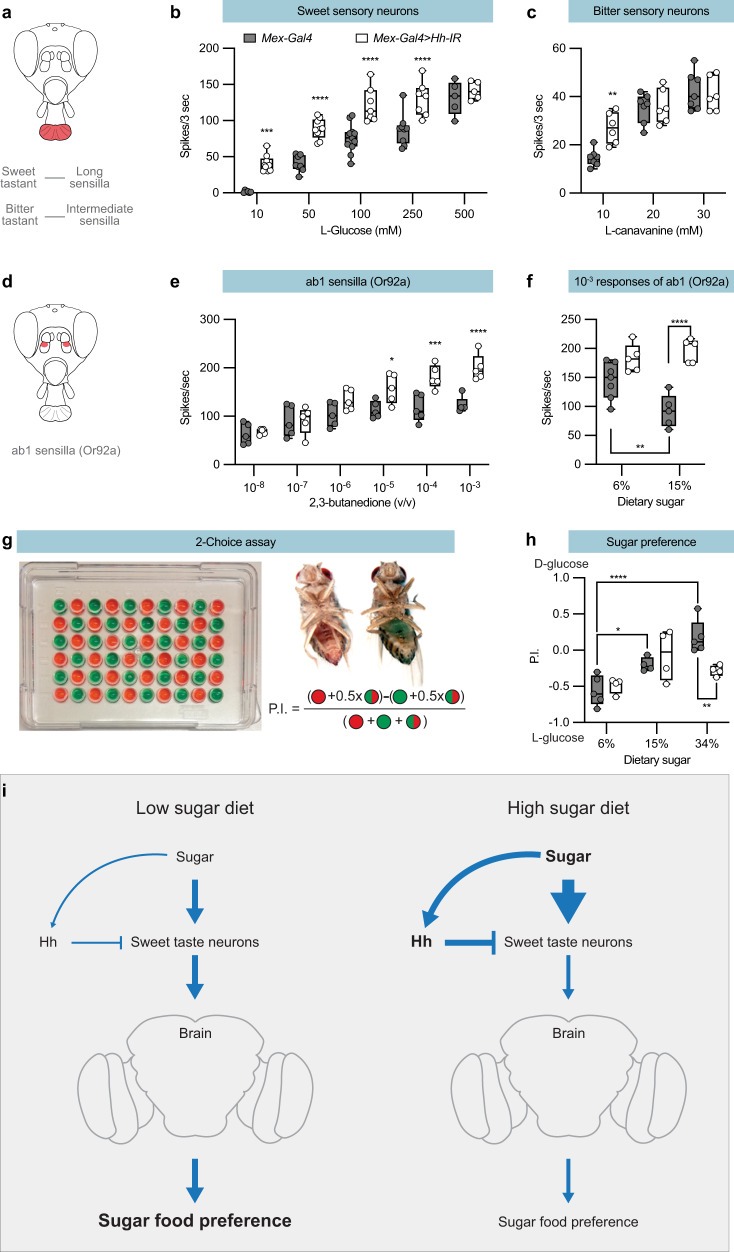
Gut Hh suppresses taste and odour responses, as well as food preference. **a** Schematic of the *Drosophila* head, highlighting the labellum in red. Electrophysiological responses was recorded from long and intermediate sensilla stimulated by sweet and bitter substances, respectively. **b** Tip recordings from long sensilla responding to a sweet substance (L-Glucose). *Mex-Gal4*, *n* = 6, 8, 11, 9, and 5; *Mex-Gal4* > *Hh-IR*, *n* = 9, 9, 7, 7, and 5. **c** Tip recordings from intermediate sensilla responding to a bitter substance (L-canavanine). *Mex-Gal4*, *n* = 7; *Mex-Gal4* > *Hh-IR*, *n* = 6. **d** Schematic of the *Drosophila* head, highlighting the Ab1-rich region of the antenna in red. **e** Electrophysiological responses of ab1B neurons (Or92a) to 2,3-butanedione. *Mex-Gal4*, *n* = 5; *Mex-Gal4* > *Hh-IR*, *n* = 5. **f** Electrophysiological responses of ab1B neurons (Or92a) in flies reared on the 6 and 15% sugar diets to a 10^−3^ dilution of 2,3-butanedione. *Mex-Gal4*, *n* = 7 and 5; *Mex-Gal4* > *Hh-IR*, *n* = 5. **g** Schematic of the two-choice assay and the calculation of the preference index. Male flies starved for 6 h were introduced to the arena at room temperature (∼23 °C) in the dark, and their preferences (abdomen color) were scored after 2 h. **h** The sweet preference of *Mex-Gal4* > *Hh-IR* flies on the 6%, 15%, and 34% sugar diets in the two-choice assay (50 mM D-glucose versus 200 mM L-glucose). Flies bearing *Mex-Gal4* alone were used as controls. *Mex-Gal4*, *n* = 5, 4, and 5; *Mex-Gal4* > *Hh-IR*, *n* = 4. **i** Model for how the sugar gut/taste neuron axis regulates sweet perception. High sugar diet increases Hh in the circulation, which reduces sweet perception and sugar preference. More details in the text. **b**, **c**, **e**, **f**, and **h** Median (middle line) is depicted, and whiskers indicate minimum to maximum. **b**, **c**, **e**, **f** *n* = 5–11 independent sensilla recordings from 3 flies. **h** *n* = 4–5 independent repeats with 20 flies in each. Statistical significance was assessed via two-way ANOVA with the Sidak correction. *p < 0.05; **p < 0.01; ***p < 0.001; ****p < 0.0001. Source data are provided as a Source Data file.

To examine the effect of midgut Hh on taste responses, we performed tip recordings from taste sensilla. There are 3 subtypes of taste sensilla on the labellum—long, intermediate, and short— that all respond to sugars^41,42^. Recordings from long taste sensilla (Fig. 7a) showed larger sweet responses in *Mex-Gal4* > *Hh-IR* flies than in *Mex-Gal4* control flies (Fig. 7b), indicating that Hh from the midgut continuously suppresses sweet taste responses. We next asked whether midgut Hh affects bitter responses by recording from the intermediate sensilla that contain sensory neurons responding to the bitter compound L-canavanine^43–45^. In contrast to the broad regulation of sugar responses, we found an increase in the response to the lowest L-canavanine concentration in *Mex-Gal4* > *Hh-IR* flies (Fig. 7c). These data suggest Hh affects both sugar and canavanine sensation but with different concentration-response thresholds.

To determine whether ingested sugar and the midgut Hh signal can suppress odour responses, we performed single sensillum recordings. Olfactory sensory neurons (OSNs) form classes that typically express a single odorant receptor that binds a distinct set of odorants^46,47^. Recordings from midgut Hh knock-down flies and control flies (*Mex-Gal4*) showed that loss of Hh increased responses in 5 out of 8 tested OSN classes (Fig. 7d, e and Fig. S5). We next asked whether elevated dietary sugar suppresses odour responses. Compared to flies maintained on the 6% diet, those maintained on food containing 15% sucrose exhibited reduced responses in their Or92a sensory neurons (Fig. 7f). Hh knock down in the midgut abolished this suppression (Fig. 7f). These results indicate that Hh secreted from the midgut suppresses both taste and olfaction.

### Dietary sugar and midgut-derived Hh regulate sugar preference

The fact that sugar regulates sweet sensation suggested that the midgut and Hh may also regulate food choice. We therefore used a two-choice feeding assay to determine sweet preference. In this assay, we allowed flies to choose between metabolizable D-glucose and four times more L-glucose that is sweet but non-metabolizable, each labelled with a different coloured dye (Fig. 7g). Consistent with previous studies^48,49^, we found flies reared on the standard 6% sugar diet preferred the sweeter L-glucose-containing food (Fig. 7h). When the flies were maintained on a 15% sugar diet, however, their preference was lost. Flies maintained on the 34% sugar diet preferred the less sweet D-glucose-containing food, which is the opposite of the preference of flies maintained on the 6% sugar diet (Fig. 7h). Thus, sugar feeding history controls sweet preference. We next asked whether the high sugar diet induced change of food preference requires midgut Hh expression. Despite the strong effect of midgut Hh knock down on taste perception, *Mex-Gal4* > *Hh-IR* flies and control flies exhibited similar sweet preference when maintained on either the 6% or shifted to a 15% sugar diet (Fig. 7h). However, compared to controls the midgut Hh knock down flies shifted to the 34% sugar diet showed an increase in sweet preference (Fig. 7h). Thus, sugar-induced midgut-derived Hh suppresses sweet preference and regulates food choice in *Drosophila*.

## Discussion

Here, we have shown that even a slight increase in sugar intake suppresses sweet perception and food-related olfactory inputs in *Drosophila*. Flies fed a high sugar diet lose interest in sweet foods, suggesting that they assign a reduced value to sugar-rich food. Our mechanistic analysis further revealed that Hh secreted from the midgut is the sugar signal that reduces sugar responsiveness and alters the animal’s preference for sweet foods. The fact that sugar intake induces midgut Hh, connects sugar intake to sweet food preference and form a closed loop feedback. This feedback mechanism connects the animals feeding pattern with future consumption and reduces the risk of continuous overconsumption and malnutrition.

Previously reported signals that regulate the chemosensory systems of *Drosophila* originate from the brain in a top-down direction^11,50–55^. The Hh signal we have identified originates in the midgut and alters sensory inputs in a bottom-up direction, a “gut taste neuron axis”. This mechanism is distinct from the well-studied “gut-brain axis” in vertebrates. In the “gut-brain axis”, direct peptidergic outputs or vagal nerve impulses arising from the gut inform the brain’s feeding centres about the animal’s metabolic state^56^. In *Drosophila*, feeding is also regulated by enteric nervous inputs to the brain^57–60^, suggesting that the Hh-mediated regulation of food detection by the sensory system works in parallel with the “gut-brain axis”. Our results further suggest that the midgut Hh endocrine feedback does not inform the brain, it rather regulates the sensory input to the brain and might be an adaptation to minimize information flow. High gut sugar is a sign of overconsumption and indicates that sugar is in excess in the most abundant nearby food source. Therefore, suppression of sweet taste perception reduces the influx of superfluous information into the brain (Fig. 7i), limiting the need for complex filtering to keep neurotransmission in the brain within a physiological and functional range. A “gut taste neuron axis” further suggests that the body like this can modulate the feeding decisions of the brain and that the endocrine Hh signal from the body converge with and balance the brain signals in the regulation of taste and olfaction perception.

The fact that an endocrine Hh ligand can suppress canonical Hh signalling in other tissues is unorthodox. We have shown that expression of a dominant negative *Ptc*^*1130X*^ prevents high dietary sugar from suppressing the PER, suggesting that autocrine and endocrine Hh signals converge at the level of the Hh receptor Ptc. This is interesting, especially since the Eaton group that discovered the circulating Hh signal also showed that sterol-modified Hh is transported with lipoprotein particles^20^. They showed that lipoprotein-associated Hh inhibits Hh target gene expression in the wing disc, functioning almost like a dominant negative influence on the Hh pathway. Disp is required for the secretion of sterol-modified Hh^61^ into the haemolymph^20^. We showed that Disp is also required in the gut for PER suppression. This supports a model in which sterol-modified Hh from the gut associates with lipoprotein particles and then circulates to inhibit sensory neuron autocrine Hh signalling.

Surprisingly, the contrasting endocrine and autocrine Hh signals cooperate during chemosensory development resulting in an increased PER. When and how the endocrine Hh signal undergoes the switch from its cooperative developmental role to its suppressive adult function remains unclear. Our TARGET results, however, suggest that the switch occurs a short time after hatching. It is thus possible that a gradual uncoupling of the endocrine Hh from promoting sweet taste neuron function during development to a suppression of taste neuron activity in the adult prevent an overshoot of sweet taste input to the brain. Adult taste sensation is also hyper-responsive and dysfunctional without the suppressive Hh midgut signal (*Mex-Gal4* > *Hh-IR*). We have further shown that when flies eclose and begin to feed, Hh gut expression increases and oscillates with decreasing amplitude until the system finally settles two days later. Thus, the switch in endocrine function would safeguard against non-adaptive taste responses through a fine-tuning of the final step of taste and olfactory neuron development.

The fact that initial sugar intake determines the adult basal Hh expression level suggests we may have identified a bona fide critical window (sensitive period). The sugar-induced regulation of Hh expression also fulfils the criteria for a critical window^62,63^. These criteria include: (i) a restricted duration, we observe that the adult level is determined by day two post eclosion; (ii) the system is most sensitive to environmental cues during the window, in this case sugar content in the food; and (iii) the induced state, the midgut Hh expression level, becomes permanent after the window. It further remains to be identified if the midgut Hh secretion on the other hand is direct regulated by sugar and if the endocrine Hh function as a proxy for sugar intake. It also remains to be shown if the endocrine Hh regulate in parallel to insulin regulate sugar metabolism and carbohydrate uptake. Another interesting aspect of the identified feedback is if other macronutrients like fat and proteins might induce the release of signals that together modulate food perception, preference, and metabolism.

A final interesting aspect of the revealed Hh feedback is that most steps of the Hh signaling pathway are conserved to mice and humans, as is the sugar suppression of sweet taste and that the Hh pathway modulate taste and olfaction. It is thus possible that an endocrine Hedgehog signal might in parallel to insulin and the gut brain axis regulate sugar rich food intake and determine food preference also in humans.

## Methods

### Drosophila strains

The following stains were used for tissue-specific transgene expression: *Mex-Gal4* (Bloomington Drosophila Stock Center, BDSC, #91368) for midgut enterocytes, *Gr64f-Gal4* (BDSC, #57669) for sweet sensory neurons. The *Hh-T2A-Gal4* (BDSC, #67493) Trojan line was used to recapitulate the expression of endogenous *Hh*. For the TARGET experiments, *Mex-Gal4; tub-Gal80*^*ts*^ (BDSC, #7017) was used to drive expression in enterocytes. Additional lines used from the BDSC: *UAS-Hh-IR* (#32489), *UAS-ptc*^*1130X*^ (#52215), *UAS-disp-IR* (#44633), *UAS-GFP.nls* (#4776) and *w*^*1118*^ (#3605). A detailed list of the *Drosophila* strains used in this study is provided in the reporting summary and in the supplemental information (Table S1).

Ptc-GFP reporter flies. The first 830 bp upstream of the Ptc ATG with wt or mutated Ci sites were synthesized at Genescript and cloned into a transformation vector containing a synthetic TATA region fused to a single ORF that contained the mCD8 transmembrane domain, four tandem copies of GFP, and two c-*myc* epitope tags^46,64^. The DNA constructs were injected into *w*^*1118*^ flies at BestGene.

### Fly husbandry

For general handling, 50–100 virgin females were crossed with 10–20 males and maintained in bottles on the 6% standard diet. All flies were reared in a 25 °C incubator on a 12 h dark/12 h light cycle under constant 60% humidity, unless otherwise mentioned. The parental flies were flipped into new bottles or disposed of after 2–3 days. Within 12 hours of eclosion, the flies were collected and transferred dependent on experiment into fresh food vials with the defined sugar diet (22–25 flies/vial). Recipes in Table 1.

**Table 1:** Fly food recipe

1L Flyfood formula		
Agar	10 g	
Brewers yeast	80 g	
Yeast extract	20 g	
Peptone	20 g	
Sucrose	6%	61 g
	7.5%	75 g
	10%	99 g
	15%	149 g
	34%	342 g
Propionic acid	6 mL	
Nipagin	11 mL	

### Haemolymph analysis

Western blots were performed as previously described^65^. Haemolymph was collected from 4-day-old male adults. In brief, each fly was cut a small opening at the abdomen (avoid damaging the internal organs, e.g., intestine, etc) using fine-forceps or scissors. The flies were placed into in a 0.5 mL tube (20 flies/tube, small holes were pricked at the bottom) that was placed on top of a 1.5 mL tube containing 5 µl 2× SDS loading buffer. The 1.5 mL tubes were centrifuged at 1 × 10^4^ rpm for 5 min at 20 °C. The total protein was separated on 10% Bis-Tris Protein Gels and transferred onto PVDF membranes (pore size 0.45 μm, Immobilon®-P, Thermoscientific) using constant current 200 mA for 90 min. The membranes were blocked with 5% milk in 1xTBST for 2 hours at room temperature. Incubation with primary antibodies was performed at 4 °C overnight with shaking. Incubation with secondary antibodies was performed at room temperature for 2 hours. The following antibodies were used: rabbit anti-Hh (1:5000), HRP conjugated anti-rabbit (1:5000, GE). The membranes were developed with the Azure 600 imaging system. The protein levels were quantified using Fiji software and normalized against Coomassie Brilliant Blue (CBB) stained total protein on the membranes.

### Behaviour assays

Proboscis Extension Response (PER) assays were performed 4 days after the switch to the experimental diet. The flies were anesthetized on ice, mounted into 200-µL pipette tips (Cat# 89079-476, VWR) cut so that only the fly’s head was exposed, and then aligned on a glass slide. The flies were placed in a humid chamber and allowed to recover for 60–90 min. Before the assay, the flies were stimulated with water and allowed to drink until satiated. Then, the labellum was stimulated with tastants using a 200-µL pipette tip attached to a 1-mL syringe. The *Drosophila* labellum was stimulated three times for each tastant with a 1 min intertrial interval. The fly that showed full proboscis extension was recorded as 1, otherwise was recorded as 0. Thus, for each fly, the total number PER would be 0, 1, 2, or 3, and calculated as a percentage of response 0%, 33.3%, 66.7%, and 100%, respectively.

For the two choice assays, flies were maintained on the indicated diets for four days before being wet starved for 6 hr. The assays were then performed using 60-well microtiter plates with drops of either 1% agarose mixed with 200 mM L-glucose and 0.5% red dye or 50 mM D-glucose and 0.7% green dye added to alternate wells. After 120 minutes of feeding, a preference index was determined by examining the abdomen colour of each fly. Flies with brown (mixed) abdomens were assigned as mixed. The preference index (P.I.) was calculated using the following equation: P.I = (# of red abdomens + 0.5 * mixed abdomens)–(green abdomens + 0.5 * mixed abdomens) / total abdomens. An absolute preference for 50 mM D-glucose or 200 mM L-glucose would result in a PI of 1 or −1, respectively. Flies that showed no preference for either sugar had mixed colour abdomens and produced a PI of 0.

### Quantitative PCR

To quantify sugar-induced changes in midgut Hh expression, entire midguts from 25–30 adult male flies were dissected, and the Malpighian tubules and hindguts were discarded. Three to seven bioreplicates was performed per data point. The samples were directly stored on ice in RNAlater (Qiagen). After dissection, RNA was extracted with the RNeasy Mini Kit (74104, QIAGEN, USA) and transcribed into cDNA using the iScript cDNA Synthesis Kit (1708890, Bio-Rad, USA). Quantitative PCR was performed using the iTaq Universal SYBR Green Supermix (1725121, Bio-Rad, USA) in a Bio-Rad CFX Connect Real-Time PCR Detection System with Actin as a control (primer sequences, table S2). All qPCR reactions were performed in at least biological triplicate, with each time series being performed together, and the results were analysed using GraphPad Prism 9. We corrected the expression of all samples to that of actin and used an average of the corrected control samples from the experiment as reference. Relative expression was determined as 2^−ΔΔCT^. Statistical significance was assessed using Student’s *t*-tests and ANOVAs with corrections for multiple comparisons.

### Immunohistochemistry

Newly eclosed adult male flies were collected into fresh food vials (22–25 flies/vial) and placed on the indicated diets. Four days after collection, the brains or heads were either dissected or embedded and stained as previously described^46^. The following antibodies were used: mouse monoclonal anti-Elav 1:100 (DSHB, 9F8A9); mouse monoclonal anti-Nc82 1:100 (DSHB, Brp); mouse monoclonal anti-21A6 1:1000 (DSHB, Eys), Rabbit ant-Hh 1:2000^28^; chicken anti GFP 1:1000 (Abcam ab13970); donkey anti-rabbit IgG (H + L) Alexa Fluor® 488 (A21206, Invitrogen); and donkey anti-mouse IgG (H + L) Alexa Fluor® 647 1:500 (Cat# 715-605-151, Jackson ImmunoResearch). Confocal microscopy images were collected on either an LSM 700 (Zeiss) or a Leica SP8 confocal microscope, Each experiment was repeated at least three times and images processed from 6 animals. Relative fluorescence intensity in each confocal image was quantified using the Fiji software (https://imagej.net/Fiji).

### Electrophysiology

Tip recordings from labellar sensilla were performed as previously described^66,67^. Briefly, 8–10-day-old flies were immobilized in pipette tips with their labellum fixed in a stable position on a glass coverslip. A glass capillary filled with Ringer’s solution (140 mM NaCl, 3 mM MgCl_2_, 2 mM CaCl_2_, 10 mM D-glucose, 10 mM HEPES, pH 7.4) was connected to an amplifier through a silver wire and inserted into the thorax of the fly. The tastants were dissolved in 30 mM tricholine citrate (TCC), an electrolyte that inhibits the water neuron. The different sensilla (long and intermediate types) were stimulated by placing a glass capillary filled with the different tastants on the sensillum tip. Sensilla on both sides of the labellum were tested. The recording electrode was connected to a pre-amplifier (TastePROBE, Syntech, Hilversum, The Netherlands), and the signals were collected and amplified (10x) using a signal-connection interface box (Syntech) in conjunction with a 100–3000 Hz band-pass filter. Action potential measurements were acquired with a 9.6 kHz sampling rate and analyzed with AutoSpike. Responses were quantified by counting the number of spikes generated during a 3-second period after contact. Responses to the TCC diluent were subtracted. Single sensillum recordings (SSR) from antennal sensilla were performed as previously described^68^. Briefly, adult flies were immobilized in pipette tips, and the third antennal segment was stabilized on a glass coverslip. Sensilla types were localized under a microscope (BX51WI; Olympus) at 100x magnification. Extracellular signals originating from the OSNs were recorded via a sharpened tungsten wire electrode inserted into the base of a single sensillum and a reference electrode inserted into the eye. Signals were amplified (Syntech Universal AC/DC Probe; Syntech), sampled (10,667.0 samples/s), and filtered (300–3,000 Hz with 50/60 Hz suppression) via a USB-IDAC connection to a computer (Syntech). Action potentials were extracted using AutoSpike software, version 3.7 (Syntech). Synthetic compounds were diluted in dichloromethane (DCM, Sigma-Aldrich, Steinheim, Germany). Prior to each experiment, 10 μl of diluted odour was freshly loaded onto a small piece of filter paper (1 cm^2^, Whatman, Dassel, Germany), and placed inside a glass Pasteur pipette. The odorant was delivered by placing the tip of the pipette 2 cm away from the antennae. Neuron activities were recorded for 10 s, starting 2 s before a stimulation period of 0.5 s. Responses from individual neurons were calculated as the increase (or decrease) in the action potential frequency (spikes/s) relative to the pre-stimulus frequency. Traces were processed by sorting spike amplitudes using AutoSpike, analyzed and graphed using Excel, and arranged into figures in Adobe Illustrator CS (Adobe systems, San Jose, CA).

### Data and statistical analysis

Data plotting and statistical analyses were performed using GraphPad Prism. Data normality was tested via the Shapiro-Wilk test. Normally distributed data were analysed via Student’s t-tests with Welch’s correction (two groups) or via one-way ANOVAs with Dunnett’s correction or two-way ANOVA with Sidak’s correction. Non-normally distributed data were analysed via either two-tailed Mann-Whitney tests (two groups) or Kruskal-Wallis H-tests with Dunn’s tests for multiple comparisons. Box plots show the median and the first and third quartile, with whiskers indicating the full range of values (except whiskers indicating Tukey in Fig. 1c). No data were excluded. Sample-size calculations were not performed. Instead, sample size was chosen on the basis of similar previously published studies of Drosophila behaviour and metabolism^52,69^.

### Reporting summary

Further information on research design is available in the Nature Portfolio Reporting Summary linked to this article.

## Supplementary information

Supplementary Information

Reporting Summary

## Data Availability

All data generated or analysed during this study are available as Source Data files, which are provided with this paper. Source data are provided with this paper.

## Source data

Source Data
