# Supplementary material for: Hedgehog-mediated gut-taste neuron axis controls sweet perception in Drosophila
Source: Nat Commun. 2022 Dec 19;13:7810. doi: 10.1038/s41467-022-35527-4 (PMC9763350; doi:10.1038/s41467-022-35527-4)
Supplement: Supplementary file 1 — Supplementary Information [file 41467_2022_35527_MOESM1_ESM.pdf]

Supplementary Information

Hedgehog-mediated gut-taste neuron axis controls sweet perception in *Drosophila*

Yunpo Zhao[1,7], Mohammed A. Khallaf [2, 3,4], Emilia Johansson[1], Najat Dzaki[1], Shreelatha Bhat[1], Johannes Alfredsson[5, 6], Jianli Duan[1,7], Bill S. Hansson[2], Markus Knaden[2] and Mattias Alenius[1,]

1. Department of Molecular Biology, Umeå University, Umeå, Sweden

2. Max Planck Institute for Chemical Ecology, Jena, Germany

3. Department of Zoology and Entomology, Faculty of Science, Assiut University, Assiut, Egypt

4. Current address: Department of Neuroscience, Max Delbrück Center for Molecular Medicine, Berlin, D-13122, Germany

5. Department of Biomedical and Clinical Sciences, Linköping University, Linköping, Sweden

6. Current address: Department of Microbiology and Immunology, Gothenburg university, Gothenburg, Sweden

7. Current address: School of Medicine, University of Maryland, Baltimore, USA

* mattias.alenius@umu.se

Supplementary Figures 1-5 and Supplementary Table 1-2

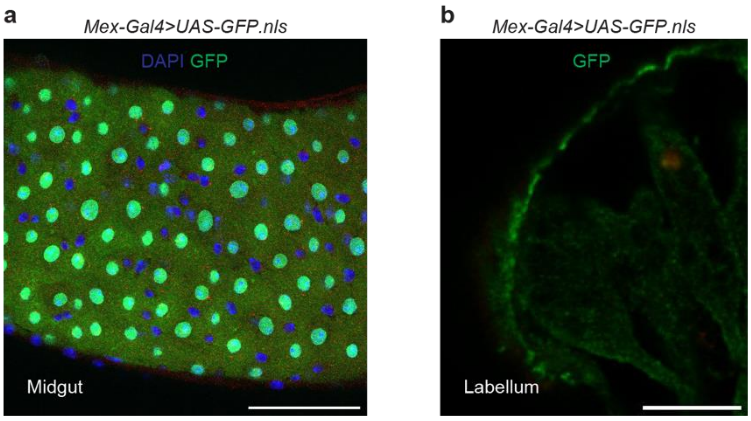

Supplementary Figure S1 ***Mex-Gal4*** **expression is not detected in the labellum.**

a Confocal image of an adult midgut (posterior part). *Mex-Gal4* drives nucleus localized GFP (GFP.nls) expression in the polyploid enterocytes. DAPI stains DNA blue. Scale bar, 50 µm. **b** Confocal image of *Mex-Gal4>UAS-GFP.nls* labellum. Scale bar, 20 µm.

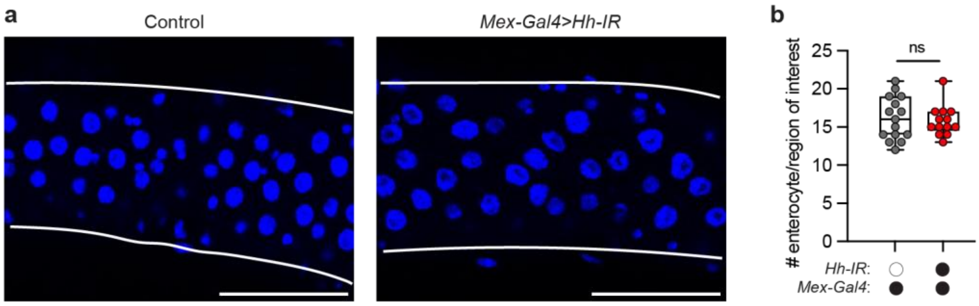

Supplementary Figure S2 ***Mex-Gal4>Hh-IR*** **does not have a strong effect on enterocyte number.**

a Representative confocal image of adult midguts from control (left) and *Mex-Gal4>Hh-IR* flies (right). DAPI staining appears in blue. Scale bars, 50 µm. **b** A box plot showing enterocyte number per region of interest in the midgut. No significant difference was observed between control and *Mex-Gal4>Hh-IR* flies. Median (middle line) is depicted, and whiskers indicate minimum to maximum. *n* = 15 and 13, respectively. Statistical analysis was performed via two-tailed *t*-test.

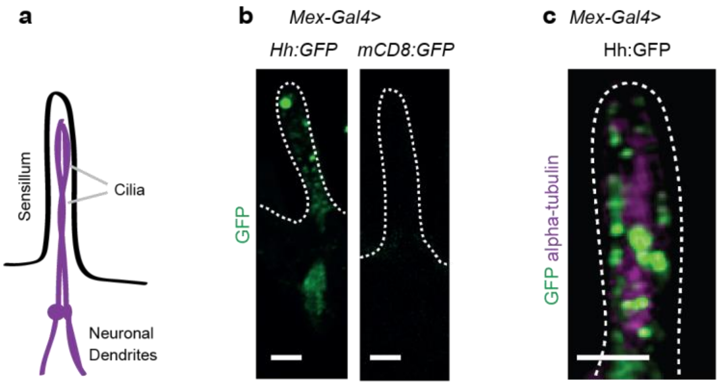

Supplementary Figure S3 Hh:GFP but not mCD8:GFP is transported from the gut to the olfactory sensillum.

a Schematic illustration of cilia. **b** Representative confocal images of adult olfactory sensilla. GFP fluorescence is shown in green. Left, *Mex-Gal4>Hh:GFP*; Right, *Mex-Gal4>mCD8:GFP*. **c** Confocal image of an adult olfactory sensillum (*Mex-Gal4>Hh:GFP*). GFP fluorescence is shown in green. Anti-alpha-Tubulin is shown in magenta. Scale bars 1 μm.

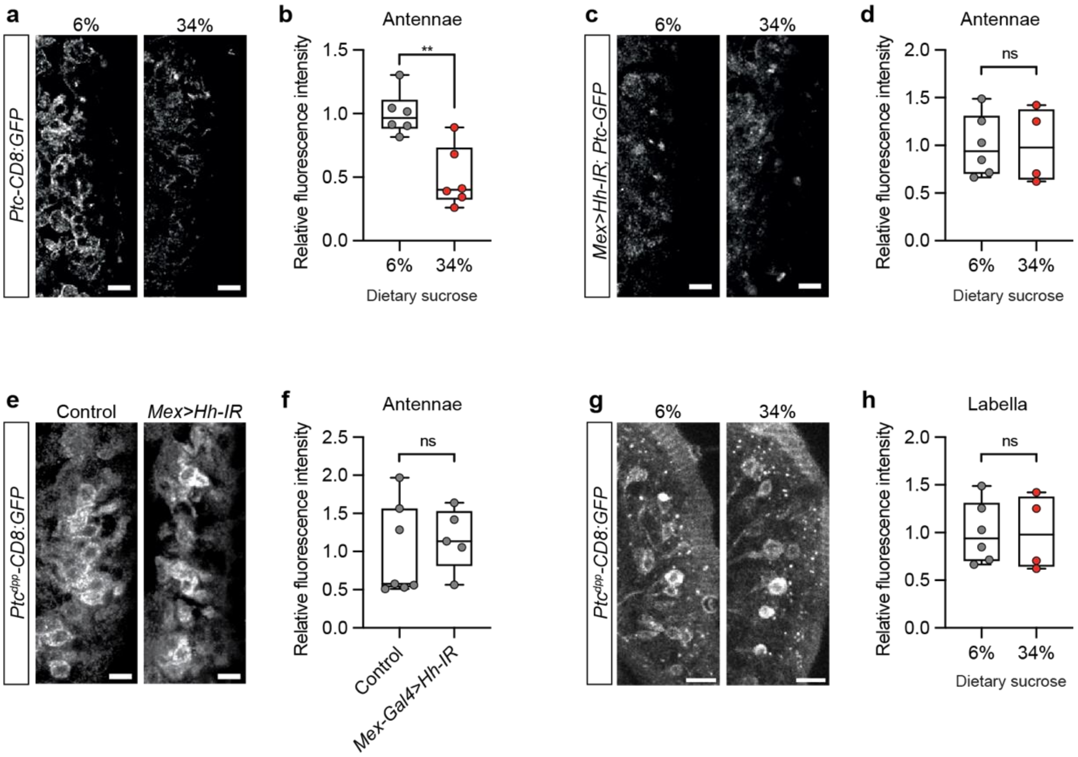

Supplementary Figure S4 Sugar-induced Hh supress Ptc expression in olfactory sensory neurons.

a Representative confocal image of adult olfactory sensilla in *ptc-CD8:GFP* flies maintained on the 6% and 34% sugar diets, respectively. GFP is shown in grey. **b** Quantification of relative ptc-CD8:GFP expression in a. $n = 6$. **c** Representative confocal images of adult olfactory sensilla of *Mex-Gal4>Hh-IR* flies maintained on the 6% and 34% sugar diets. **d** Quantification of relative ptc-CD8:GFP expression in c. $n = 6$ and 4, respectively. **e** Confocal image of adult olfactory sensilla of *ptc^{dpp}-mCD8:GFP* flies. This reporter has a mutation in its Ci binding motifs, rendering it insensitive to Hh signalling. **f** Quantification of the relative fluorescence intensity of ptc^{dpp}-mCD8:GFP expression in e. Left, control (*Mex-Gal4*); right, *Mex-Gal4>Hh-IR*. $n = 7$ and 5, respectively. **g** Confocal image of adult labella from *ptc^{dpp}-mCD8:GFP* flies maintained on the 6% and 34% sugar diets. **h** Quantification of the relative fluorescence intensity of ptc^{dpp}-mCD8:GFP expression in g. $n = 6$ and 4, respectively. Scale bars, 5 µm (a, c, and e) or 10 µm (g). Median (middle line) is depicted, whiskers represent minimum to maximum (b, d, f, and h). Statistical analyses were performed via two-tailed *t*-test with the Welch correction. **, $p < 0.01$.

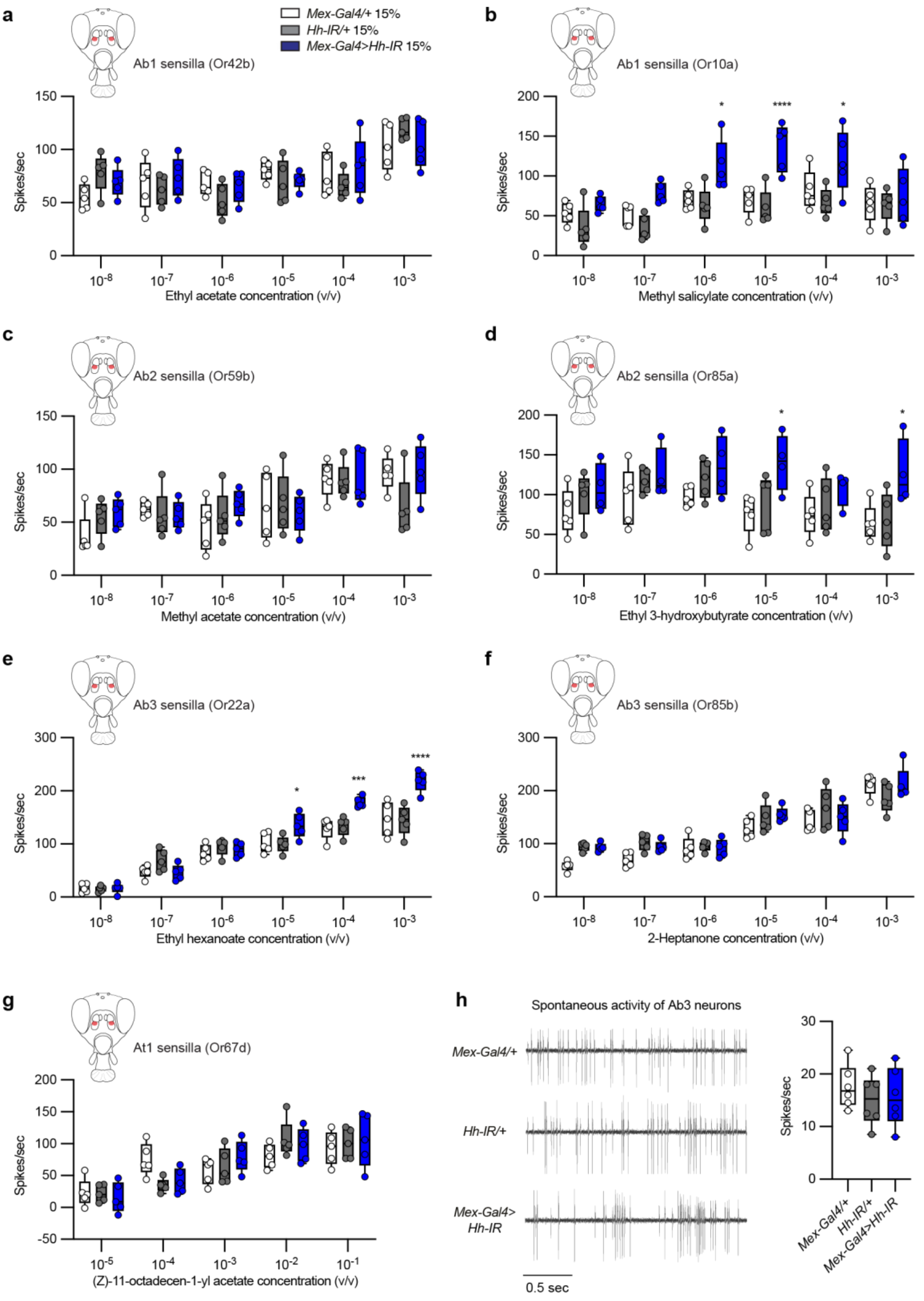

Supplementary Figure S5 Midgut Hh regulates odour responses.

a Electrophysiological responses of ab1A neurons (Or42B) to ethyl acetate. *n* = 5. **b** Electrophysiological responses of ab1D neurons (Or10a) to methyl salicylate. *n* = 5. **c** Electrophysiological responses of ab2A neurons (Or85a) to methyl acetate. *n* = 5. **d** Electrophysiological responses of ab2B neurons (Or85a) to ethyl 3-hydroxybutyrate. *n* = 5. **e** Electrophysiological responses of ab3A neurons (Or22a) to ethyl hexanoate. *n* = 5, 5, and 4, respectively. **f** Electrophysiological responses of ab3B neurons (Or85b) to 2-heptanone. *n* = 5. **g**

Electrophysiological responses of at1 neurons (Or67d) to (Z)-11-octadecen-1-yl acetate. $n = 5$. **h** Left: Electrophysiological traces of the spontaneous activity of ab3 in the resting state. Right: spikes within 2 seconds. Median (middle line) is depicted, whiskers represent minimum to maximum. Statistical significance was assessed via two-way ANOVA with the Sidak correction (a-g) or one way ANOVA with the Tukey correction (h). *, $p < 0.05$; ***, $p < 0.001$; ****, $p < 0.0001$.

Supplementary Table 1: Fly lines used in this work

Flyline	Source	Identifyer
w^{1118}	Bloomington Drosophila Stock Center (BDSC)	#3605
UAS-Hh-IR	BDSC	#32489
UAS-ptc^{1130X}	BDSC	#52215
Hh-Gal4	BDSC	#67493
Gr64f-Gal4	BDSC	#57669
Mex-Gal4	BDSC	#91368
tub-Gal80ts	BDSC	#7017
UAS-disp-IR	BDSC	#44633
UAS-GFP.nls	BDSC	#4776
UAS-Hh:GFP	Isabel Guerrero, Madrid university, Madrid, Spain	
ptc-CD8:GFP	This work	
ptc^{dpp}-CD8:GFP	This work	

Supplementary Table 2: Oligo sequences used for qPCR

Primer name	Sequence (5’ to 3’)
Actin F	CACACCAAATCTTACAAAATGTGT
Actin R	AATCCGGCCTTGCACATG
Hedgehog F	CGCCAGTGTCACCTGTCTC
Hedgehog R	TTCTTGCGGGATTGCGGAG
