# Supplementary material for: Hedgehog-mediated gut-taste neuron axis controls sweet perception in Drosophila
Source: Nat Commun. 2022 Dec 19;13:7810. doi: 10.1038/s41467-022-35527-4 (PMC9763350; doi:10.1038/s41467-022-35527-4)
Supplement: Supplementary file 2 — Reporting Summary [file 41467_2022_35527_MOESM2_ESM.pdf]

nature portfolio

Corresponding author(s): Mattias Alenius

Last updated by author(s): 2022.11.09

Reporting Summary

Nature Portfolio wishes to improve the reproducibility of the work that we publish. This form provides structure for consistency and transparency in reporting. For further information on Nature Portfolio policies, see our Editorial Policies and the Editorial Policy Checklist.

Statistics

For all statistical analyses, confirm that the following items are present in the figure legend, table legend, main text, or Methods section.

n/a	Confirmed	
☐	☒	The exact sample size (*n*) for each experimental group/condition, given as a discrete number and unit of measurement
☐	☒	A statement on whether measurements were taken from distinct samples or whether the same sample was measured repeatedly
☐	☒	The statistical test(s) used AND whether they are one- or two-sided<br>*Only common tests should be described solely by name; describe more complex techniques in the Methods section.*
☒	☐	A description of all covariates tested
☐	☒	A description of any assumptions or corrections, such as tests of normality and adjustment for multiple comparisons
☐	☒	A full description of the statistical parameters including central tendency (e.g. means) or other basic estimates (e.g. regression coefficient) AND variation (e.g. standard deviation) or associated estimates of uncertainty (e.g. confidence intervals)
☐	☒	For null hypothesis testing, the test statistic (e.g. *F*, *t*, *r*) with confidence intervals, effect sizes, degrees of freedom and *P* value noted<br>*Give P values as exact values whenever suitable.*
☒	☐	For Bayesian analysis, information on the choice of priors and Markov chain Monte Carlo settings
☒	☐	For hierarchical and complex designs, identification of the appropriate level for tests and full reporting of outcomes
☒	☐	Estimates of effect sizes (e.g. Cohen's *d*, Pearson's *r*), indicating how they were calculated

Our web collection on statistics for biologists contains articles on many of the points above.

Software and code

Policy information about availability of computer code

Data collection	AutoSpike software, version 3.7 (Syntech)
Data analysis	FIJI/ImageJ (https://imagej.net/Fiji/Downloads)<br>GraphPad Prismv version 8.2 (https://www.graphpad.com/scientific-software/prism/)<br>Adobe Illustrator CS6 version (https://www.adobe.com/)

For manuscripts utilizing custom algorithms or software that are central to the research but not yet described in published literature, software must be made available to editors and reviewers. We strongly encourage code deposition in a community repository (e.g. GitHub). See the Nature Portfolio guidelines for submitting code & software for further information.

Data

Policy information about availability of data

All manuscripts must include a data availability statement. This statement should provide the following information, where applicable:

- Accession codes, unique identifiers, or web links for publicly available datasets
- A description of any restrictions on data availability
- For clinical datasets or third party data, please ensure that the statement adheres to our policy

All data generated or analysed during this study are included in this published article (and its supplementary information files).

Human research participants

Policy information about studies involving human research participants and Sex and Gender in Research.

Reporting on sex and gender	No human studies were perfromed
Population characteristics	No human studies were perfromed
Recruitment	No human studies were perfromed
Ethics oversight	No human studies were perfromed

Note that full information on the approval of the study protocol must also be provided in the manuscript.

Field-specific reporting

Please select the one below that is the best fit for your research. If you are not sure, read the appropriate sections before making your selection.

[x] Life sciences [ ] Behavioural & social sciences [ ] Ecological, evolutionary & environmental sciences

For a reference copy of the document with all sections, see nature.com/documents/nr-reporting-summary-flat.pdf

Life sciences study design

All studies must disclose on these points even when the disclosure is negative.

Sample size	For behavior assays, sample size (20-40 flies per genotype or per treatment) was chosen according to previous publications. Chu, B., V. Chui, K. Mann, and M. D. Gordon. 2014. “Presynaptic Gain Control Drives Sweet and Bitter Taste Integration in Drosophila.” Current Biology: CB 24 (17): 1978–84. LeDue, E. E., K. Mann, E. Koch, B. Chu, R. Dakin, and M. D. Gordon. 2016. “Starvation-Induced Depotentiation of Bitter Taste in Drosophila.” Current Biology: CB 26 (21): 2854–61. The numbers of samples are large enough to capture normal variation and capture small variations
Data exclusions	No data were excluded from the analyses.
Replication	We performed two independent experiments. The attempts at replication were successful.
Randomization	Animals were random grouped into batches.
Blinding	The typical limited number of persons with expertise in these advanced studies require that setting up, handling and scoring is done by the same person. Therefore, studies were performed non blind as is praxis for fly behaviour tests.

Reporting for specific materials, systems and methods

We require information from authors about some types of materials, experimental systems and methods used in many studies. Here, indicate whether each material, system or method listed is relevant to your study. If you are not sure if a list item applies to your research, read the appropriate section before selecting a response.

Materials & experimental systems

n/a	Involved in the study	
[ ]	[x]	Antibodies
[x]	[ ]	Eukaryotic cell lines
[x]	[ ]	Palaeontology and archaeology
[ ]	[x]	Animals and other organisms
[x]	[ ]	Clinical data
[x]	[ ]	Dual use research of concern

Methods

n/a	Involved in the study	
[x]	[ ]	ChIP-seq
[x]	[ ]	Flow cytometry
[x]	[ ]	MRI-based neuroimaging

Antibodies

Antibodies used	Rabbit anti-Hh gift from Natalie Dye<br>Mouse monoclonal anti-ELAV (DSHB)<br>Mouse monoclonal anti-Nc82 (DSHB)<br>Mouse monoclonal anti-21A6 (DSHB)

	Donkey anti-rabbit IgG (H+L) Alexa Fluor 488 (A21206, Invitrogen)<br>Donkey anti-mouse IgG (H+L) Alexa Fluor 647 (Cat# 715-605-151, Jackson ImmunoResearch)<br>HRP conjugated Goat anti-Rabbit IgG (H+L) (# 31466, Invitrogen)
Validation	Rabbit anti-Hh is validated in: Rodenfels, J., O. Lavrynenko, S. Ayciriex, J. L. Sampaio, M. Carvalho, A. Shevchenko, and S. Eaton. 2014. “Production of Systemically Circulating Hedgehog by the Intestine Couples Nutrition to Growth and Development.” Genes & Development 28 (23): 2636–51.<br>Mouse monoclonal anti-ELAV (DSHB) validated in Moses K "Wingless and patched are negative regulators of the morphogenetic furrow and can affect tissue polarity in the developing Drosophila compound eye." Development 121.8 (1995 Aug): 2279-89.<br>Mouse monoclonal anti-Nc82 (DSHB) validated in Buchner E "Bruchpilot, a protein with homology to ELKS/CAST, is required for structural integrity and function of synaptic active zones in Drosophila." Neuron 49.6 (2006 Mar 16): 833-44.<br>Mouse monoclonal anti-21A6 (DSHB) validated in Kuzhandaivel, A., Schultz, S.W., Alkhori, L and Alenius M. 2014 "Cilia-Mediated Hedgehog Signaling in Drosophila" Cell reports<br>Donkey anti-rabbit IgG Alexa Fluor 488, validated by manufacturer (https://www.thermofisher.com/antibody/product/Donkey-anti-Rabbit-IgG-H-L-Highly-Cross-Adsorbed-Secondary-Antibody-Polyclonal/A-21206)<br>Donkey anti-mouse Alexa Fluor 647 validated by manufacturer. (https://www.jacksonimmuno.com/catalog/products/715-605-151)<br>HRP conjugated Goat anti-Rabbit validated by manifacturer (https://www.thermofisher.com/antibody/product/Goat-anti-Rabbit-IgG-H-L-Secondary-Antibody-Polyclonal/31460)

Animals and other research organisms

Policy information about studies involving animals; ARRIVE guidelines recommended for reporting animal research, and Sex and Gender in Research

Laboratory animals	This study made use of a varaiety of stocks of Drosophila Melanogaster, detailed in the manuscript. Stocks origin bellow:<br><br>UAS-Hh:GFP kind gift from Isabel Guerrero.<br><br>Flies obtained from the University of Indiana Bloomington Drosophila Stock Center:<br>w1118 #3605<br>Mex-Gal4 #91368 (original a kind gift from Irene Miguel-Aliaga)<br>Gr64f-Gal4 #57669<br>Hh-Gal4 #67493<br>UAS-Hh-IR #32489<br>UAS-ptc1130X #52215<br>UAS-disp-IR #44633<br>tub-Gal80ts #7017<br>UAS-GFP.nls #4776<br><br>Flies generated for this work:<br>ptc-CD8:GFP<br>ptc(Dpp)-CD8:GFP
Wild animals	No wild animals were used in this study.
Reporting on sex	All animals in this study were male flies.
Field-collected samples	No field collected animals were used in this study
Ethics oversight	No ethics approval or oversight is required for Drosophila studies.

Note that full information on the approval of the study protocol must also be provided in the manuscript.
